# Urological activity at the time of COVID-19 pandemic: is there any difference between public and private field?

**DOI:** 10.11604/pamj.2020.37.389.25297

**Published:** 2020-12-31

**Authors:** Skander Zouari, Ahmed Saadi, Marouene Chakroun, Amine Oueslati, Maroua Fliss, Abderrazek Bouzouita, Amine Derouiche, Riadh Ben Slama, Haroun Ayed, Mohamed Chebil

**Affiliations:** 1 Tunis El Manar University, Faculty of Medicine of Tunis, Charles Nicolle Hospital, Department of Urology, Tunis, Tunisia

**Keywords:** COVID-19, pandemics, urology, urological activity, impact

## Abstract

**Introduction:**

as COVID-19 pandemic is rapidly evolving, there is a whole reorganization in hospitals to concentrate more resources to face the crisis. The purpose of this study is to evaluate the impact of COVID-19 disease on urological activity in Tunisia. To assess the differences in the management of urological conditions between the private and the public field.

**Methods:**

a survey was addressed to all certified urologists working in Tunisia in both the public and private sectors (n=194) using the national database of active urologists available and updated. We either called them or looked them up through email or social media. The form was open from March the 28^th^ to April the 3^rd^. Results were obtained via spreadsheet and analysed using SPSS 23.0.

**Results:**

one hundred and twenty urologists have filled in the form. Consultations at the outpatient office were restricted to urgent cases in 66% (n=79). Telemedicine was more used by urologists in private than in public fields p=0.03. Urologists in private sector followed more the sterilization protocol of the hospital/clinic and used more disposable materials whenever possible p=0.011. Elective surgical activity has completely stopped in 85% of the responders (n=102). Elective surgery requiring transfusion or intensive care unit was performed in 38% (n=46) and 26% (n=31) if there was a risk of disease progression. Benign Prostate Hyperplasia (BPH) surgery was more performed as usual in private sector than in public sector p=0.012. It was the only condition managed differently between both sectors.

**Conclusion:**

the drop of the urological activity is essential in order to give relevant stakeholders room to act efficiently against the spread of the virus. The context of the pandemic and the hospital´s condition must be taken into consideration without compromising the patient´s outcome.

## Introduction

Coronavirus disease 2019 (COVID-19) is an infectious disease that causes severe acute respiratory syndrome, coronavirus 2 (SARS-CoV-2). It was first discovered in Wuhan, China, where pneumonia of an unknown cause was detected. COVID-19 was reported to the WHO Country Office in China on the 31^st^ of December 2019 [1]. Since then, it has widely spread around the world. On March the 11^th^, COVID-19 was identified by the WHO as a pandemic [2]. On April 2^nd^, the world reached the threshold of one million confirmed cases. Currently, there are more than 54.6 million cases around the world with more than a million and three hundred thousand deaths. China and Italy were the first countries to be hit by the disease. Today, no region of the world is spared by the pandemic. In Africa, we count today nearly 2 million cases with 47,000 deaths. Some countries are seeing a growing number of newly confirmed COVID-19 cases like while others have flattened or inverted the curve of the disease. Perhaps simple protective measures like hand washing, social distancing and the global fear caused by the pandemic has led African countries to reduce the number of infected cases and avoid the wave of the disease expected [3].

On the 15^th^ of November, Tunisia had 80,404 confirmed cases of COVID-19, which include 2345 deaths and 282 cases in critical situation. The country did manage the first wave of the pandemic quiet well with a several restrictive measures, but was severely hit by second wave. Currently, the country is struggling with the shortage of intensive care beds and respiratory devices to host new cases of COVID-19 patients. This whole reorganization of hospitals and wards had a great impact on urological activities. It is mainly limited to non-deferrable and urgent procedures. Because there is no clear definition of what an ‘elective’ surgery is, considerations for the triage of urological procedures are between the hands of the urologist. Some authors tried to come up with recommendations in the triage of urological surgeries during the pandemic [4]. This study aimed to evaluate the impact of COVID-19 disease on urological activity in Tunisia. To assess the differences in the management of urological conditions between the private and the public field.

## Methods

**Study design and setting:** an online questionnaire was used for the purpose of the study and sent to the participants. It was open from March 28^th^ to April 3^rd^ 2020. It was composed of 12 questions assessing the impact of COVID-19 pandemic on both outpatient clinic activity and surgical activity. Urologists were asked about their attitude about urologic emergencies, sterilisation of surgical equipment, elective surgeries that requires transfusion or intensive care unit. Management of each of these following urological conditions was evaluated (prostatic biopsy, cystoscopy, removal of double J stent, benign prostate hyperplasia surgery, urolithiasis surgery, transurethral resection of bladder tumour, radical cystectomy, radical nephrectomy, radical nephroureterectomy, radical prostatectomy, urethral stricture surgery and surgery of benign conditions as hydrocele or varicocele): they could either categorize them as deferred, performed as usual, included in a surgical priority list, done in outpatient office or referred to another centre. They also rated from zero to ten each of the conditions mentioned above according to their level of priority. They were finally asked about the reasons behind the drop of the urological activity and the means to keep up with theoretical learning during the pandemic.

**Study population:** a survey was addressed to all certified urologists currently working in Tunisia in both the public and private sectors (n=194) using the national database of active urologists available and updated. They were reached either using email or phone numbers.

**Data collection and statistical analysis:** responses were collected via email, and analysed using SPSS 23.0. Chi-squared test was used to analyse data. Significance was set at p<0.05. Confidentiality and anonymity of the responses was kept during the study.

**Ethical considerations:** confidentiality of the data and anonymity of the responders were respected during the study.

## Results

**General characteristics:** one hundred and twenty urologists (62%) filled in the form, out of 193 who received the survey. Sixty three (52.5%) were aged between 40 and 50. Eighty-one (67.5%) work in the private sector. Fifty one (42.5%) were located in the capital city, Tunis.

**Figure 1:**
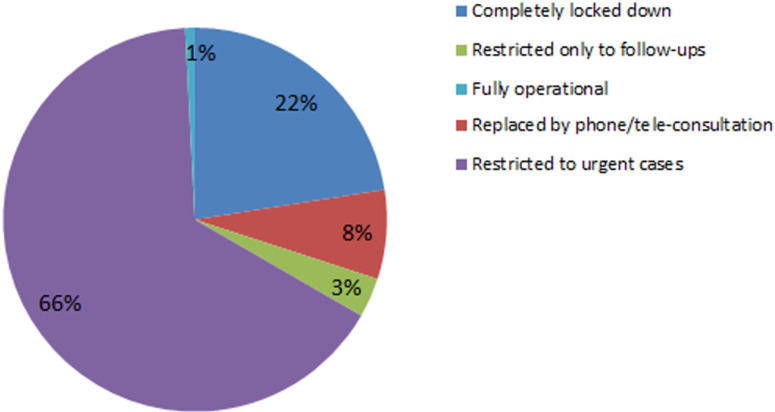
management of outpatient clinic according to Tunisian urologists

**Management of the outpatient office:** at the time of the investigation, the consultations at the outpatient office were restricted to urgent cases in 66% (n=79) (Figure 1). It was most lockdown in urologists from the public field (38.5% vs 14.8%), and most restricted to urgent cases most in urologists from the private field (71.6% vs 53.8%). Telemedicine was used more by urologists from the private field (9.9% vs 2.6%). This difference in management of outpatient clinic was statistically significant p=0.03.

**Figure 2:**
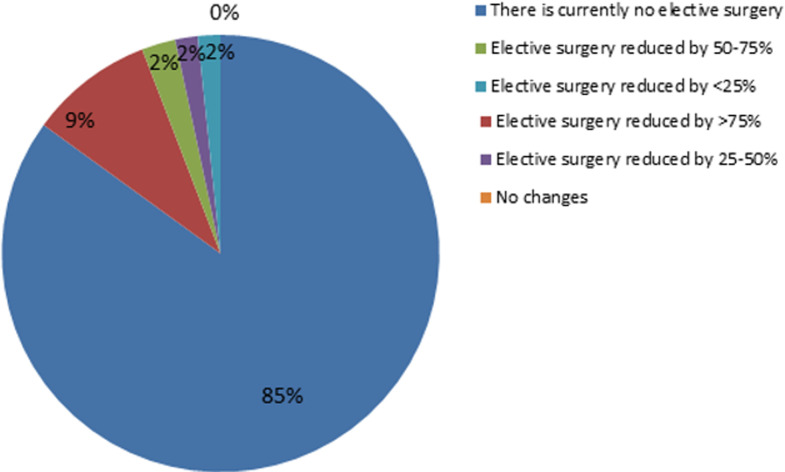
impact of COVID-19 on surgical activity among Tunisian urologists

**Management of surgical activity and surgical conditions:** in the case of an emergency that requires immediate surgery, 61% (n=73) of the urologists opted for an increased attention without specific measures while 24% (n=29) did the COVID-19 test only if the patient was symptomatic. For surgical equipment sterilization, 53.9% (n=21) of the urologists from public field didn´t take specific measures versus 25.9% (n=21) from private field. Those in private sector followed more the sterilization protocol of the hospital/clinic (38.3% vs 20.5%) or used disposable materials whenever possible (32.1% vs 17.9%). The difference was statistically significant p=0.011. Eighty-five per cent (n=102) of urologists stopped elective surgery completely (Figure 2). Seventy-six per cent of urologists (n=91) were responsible of selecting the cases that must be operated in the department they belong to with a significant difference in favour of urologists working in private sector (85.2% vs 56.4%) p < 0.001. In urologists working in public sector, they were more following the directives of the department head (30.8% vs 2.5%) or the direction of the hospital/clinic (10.2% vs 7.4%).

As of elective surgery requiring transfusion, 57% (n=68) postponed it, while 38% (n=46) performed it if there was a risk of disease progression. Seventy per cent of urologists (n=84) deferred elective surgery that required a hospitalization in intensive care unit while 26% (n=31) performed it if patients were at risk of disease progression. Main attitude towards the management of non-oncological procedures was to postpone by both urologists from public and private field (Table 1). BPH was the only urological condition that was managed differently between urologists in private and in public field p=0.012, as 100% (n=39) of urologists in public sector postpone it while it is postponed in 87.6% (n=71) of urologists working in private sector. Regarding oncological procedures, opinions diverged as a part of urologists tend to perform oncological procedures as usual, another prefer to delay them while a third part include them in a surgical priority list and perform it when the planning allows it. No significant difference has been detected between management of oncological conditions between urologists working in public and private sectors (Table 1). Attitudes were the way when urologists were asked to rate the priority of each of the aforementioned elective surgeries of 10 (Table 2). Benign conditions like hydrocele or varicocele were rated the lowest (1.24) while radical orchiectomy was rated the highest (5.54).

**Table 1:** management of urological procedures by public and private sector urologists in Tunisia during the COVID-19 pandemic

		Deferred N (%)	Performed as usual N (%)	Outpatient procedure N (%)	Included in a surgical priority list N (%)	Referred to another center N (%)	p
Prostatic biopsy	**Public sector**	35 (98.7)	2 (5.1)	2 (5.1)	0	0	0.274
	Private sector	71 (87.6)	6 (7.4)	1 (1.2)	2 (2.5)	1 (1.2)	
Cystoscopy	**Public sector**	33 (84.6)	1 (2.56)	4 (10.26)	1 (2.56)	0	0.633
	Private sector	65 (80.2)	9 (11.1)	4 (4.9)	3 (3.7)	0	
Double J stent removal	**Public sector**	30 (76.9)	3 (7.7)	5 (12.8)	0	1 (2.56)	0.701
	Private sector	58 (71.6)	11 (13.6)	7 (8.6)	5 (6.2)	0	
BPH surgery	**Public sector**	39 (100)	0	0	0	0	**0.012**
	Private sector	71 (87.6)	7 (8.6)	1 (1.2)	2 (2.5)	0	
TURBT	**Public sector**	20 (51.3)	11 (28.21)	0	8 (20.5)	0	0.257
	Private sector	27 (33.3)	37 (45.7)	1 (1.2)	16 (19.75)	0	
Urolithiasis surgery	**Public sector**	37 (94.9)	2 (5.13)	0	0	0	0.909
	Private sector	72 (88.9)	7 (8.6)	1 (1.2)	1 (1.2)	0	
Radical prostatectomy	**Public sector**	35 (89.7)	2 (5.13)	0	2 (5.13)	0	0.671
	Private sector	59 (72.8)	11 (13.6)	1 (1.2)	7 (8.6)	3 (3.7)	
Partial / Radical nephrectomy	**Public sector**	35 (89.7)	2 (2.5)	0	2 (2.5)	0	0.754
	Private sector	43 (53.1)	18 (22.2)	2 (2.5)	15 (18.5)	3 (3.7)	
Radical nephroureterectomy	**Public sector**	23 (59)	9 (23.1)	1 (2.6)	6 (15.4)	0	0.333
	Private sector	35 (43.2)	25 (30.9)	2 (2.5)	16 (19.7)	3 (3.7)	
Radical cystectomy	**Public sector**	20 (51.3)	11 (28.2)	1 (2.6)	7 (17.9)	0	0.124
	Private sector	38 (46.9)	23 (28.4)	2 (2.5)	15 (18.5)	3 (3.7)	
Radical orchiectomy	**Public sector**	16 (41)	15 (38.5)	1 (2.6)	6 (15.4)	1 (2.6)	0.453
	Private sector	26 (32.1)	34 (42)	2 (2.5)	17 (21)	2 (2.5)	
Urethral stricture surgery	**Public sector**	38 (97.4)	1 (2.6)	0	0	0	0.123
	Private sector	69 (85.2)	8 (9.9)	2 (2.5)	1 (1.2)	1 (1.2)	
Benign conditions	**Public sector**	39 (100)	0	0	0	0	0.443
	Private sector	76 (93.8)	4 (4.9)	1 (1.2)	0	0	

BPH: Benign Prostate Hyperplasia; TURBT: Trans Urethral Resection of Bladder Tumour

**Table 2:** priority given to each urological procedure according to practicing urologists

	1 (Lowest priority) N (%)	2N (%)	3 N(%)	4 N (%)	5 N (%)	6 N (%)	7 N (%)	8 N (%)	9 N (%)	10 (Highest priority) N (%)	Don't know N (%)	Average rate
Prostatic biopsy	**81 (67.5)**	**15 (12.5)**	**7 (5.8)**	**3 (2.5)**	**3 (2.5)**	**3 (2.5)**	**0**	**3 (2.5)**	**1 (0.8)**	**1 (0.8)**	**3 (2.5)**	**1.88/10**
Cystoscopy	63 (52.5)	19 (15.8)	14 (11.6)	5 (4.2)	5 (4.2)	4 (3.3)	3 (2.5)	1 (0.8)	1 (0.8)	2 (1.5)	3 (2.5)	2.31/10
Removal of the double J stent	**58 (48.3)**	**28 (23.3)**	**15 (12.5)**	**3 (2.5)**	**7 (5.8)**	**3 (2.5)**	**1 (0.8)**	**0 (0)**	**1 (0.8)**	**1 (0.8)**	**3 (2.5)**	**2.63/10**
BPH surgery	73 (60.8)	22 (18.3)	11 (9.1)	5 (4.2)	3 (2.5)	2 (1.5)	0 (0)	1 (0.8)	0 (0)	1 (0.8)	2 (1.5)	1.88/10
TURBT	**16 (13.3)**	**26 (21.6)**	**8 (6.6)**	**5 (4.2)**	**4 (3.3)**	**5 (4.2)**	**8 (6.6)**	**12 (10)**	**10 (8.3)**	**24 (20)**	**2 (1.5)**	**5.48/10**
Urolithiasis surgery	56 (46.6)	27 (22.5)	14 (11.6)	5 (4.2)	8 (6.6)	1 (0.8)	3 (2.5)	0 (0)	0 (0)	4 (3.3)	2 (1.5)	2.37/10
Radical prostatectomy	**50 (41.6)**	**15 (12.5)**	**12 (10)**	**6 (5)**	**5 (4.2)**	**7 (5.8)**	**5 (4.2)**	**6 (5)**	**6 (5)**	**5 (4.2)**	**3 (2.5)**	**3.38/10**
Radical/Partial nephrectomy	31 (25.8)	19 (15.8)	10 (8.3)	6 (5)	7 (5.8)	14 (11.6)	4 (3.3)	9 (7.5)	8 (6.6)	9 (7.5)	3 (2.5)	4.33/10
Radical Nephroureterectomy	**23 (19.1)**	**21 (17.5)**	**6 (5)**	**5 (4.2)**	**5 (4.2)**	**8 (6.6)**	**6 (5)**	**12 (10)**	**15 (12.5)**	**16 (13.3)**	**3 (2.5)**	**5.23/10**
Radical cystectomy	25 (20.8)	17 (14.1)	5 (4.2)	8 (6.6)	4 (3.3)	7 (5.8)	4 (3.3)	10 (8.3)	15 (12.5)	21 (17.5)	4 (3.3)	5.38/10
Radical orchiectomy	**14 (11.6)**	**21 (17.5)**	**12 (10)**	**5 (4.2)**	**4 (3.3)**	**7 (5.8)**	**6 (5)**	**12 (10)**	**10 (8.3)**	**22 (18.3)**	**7 (5.8)**	**5.54/10**
Uretral stricture surgery	64 (53.3)	29 (24.1)	6 (5)	7 (5.8)	4 (3.3)	5 (4.2)	1 (0.8)	0 (0)	0 (0)	2 (1.5)	2 (1.5)	2.08/10
Surgery of benign conditions	**104 (86.6)**	**8 (6.6)**	**2 (1.5)**	**2 (1.5)**	**0 (0)**	**1 (0.8)**	**1 (0.8)**	**0 (0)**	**0 (0)**	**0 (0)**	**2 (1.5)**	**1.24/10**

BPH: Benign Prostate Hyperplasia; TURBT: Trans Urethral Resection of Bladder Tumour

**Reasons behind urological activity drop and urological learning during COVID-19 period:** a hundred and three urologists (85.8%) think urological activity has dropped because of the fear of being infected or infecting other patients or the medical/paramedical staff while 40.8% (n=49) think it is because of shortage of logistical materials and 29.2% (n=35) attribute it to the lack of human resources. Most of urologists (72.5%) (n=87) think they can be infected by COVID-19 while performing their work while 23.3% (n=28) think they can contract it in the community. A few numbers (3.3%) (n=4) feel that the danger of being infected does not exist. A large majority of urologists 80% (n=96) keep with the theoretical learning during the pandemic through internet browsing, 29.2% (n=35) through journals/books, 18.3% (n=22) through social media, 16.7% through audio/video courses (n=20) while 9.2% (n=11) use webinars.

## Discussion

**COVID-19 and outpatient clinic activity:** the COVID-19 pandemic had a major effect on urological activity across the world. On the one hand, consultation activity has dramatically dropped and reduced essentially to urological emergencies. In Singapore [5], there was a rescheduling of consultation appointments, prioritizing urgent and semi-urgent conditions as obstructive uropathy, shockwave lithotripsy for an obstructive calculi or oncological conditions. The situation is practically the same in Tunisia. The main difference is that in the public field, urological activity is following the measures dictated by the minister of health while in the private field, these considerations are under the urologist´s responsibility.

**Use of telemedicine:** recent papers stress out the importance of telemedicine and the concept of virtual urology clinic for oncological conditions as an efficient tool to replace classic urological consultation [6-8]. Connor MJ *et al*. [6] believes it could keep the cancer pathway moving without compromising neither the healthcare professional nor the patient´s condition. Dubin *et al*. [7] find lack of technological comprehension, patients´ lack of access to required technology, and reimbursement concerns to be the main barriers to use telemedicine. Perhaps these reasons apply in Tunisia. Efforts are actually deployed in order to put a proper forensic framework to exert the telemedicine in Tunisia [9].

**COVID-19 and surgical activity:** to face the decrease of the surgical activity and the workload to postpone, several authors have tried to implement new measures and came up with recommendations to help prioritize surgical procedures. In Italy, Ficarra *et al*. [10] categorized urological procedures in 4 groups, depending not only on the urological procedure, but also the availability of beds in the department, the comorbidity profile and the impact of the pandemic on the geographical area. Stensland *et al*. [4] also set up some recommendations for the triage of urological surgeries, giving the priority to oncological conditions at risk of disease progression, and replacing whenever possible an open surgery by a minimally invasive one. For example, urinary tract obstruction or infection should be drained by ureteric stents or nephrostomy tubes under local anaesthesia at first place. Ureteric stents under general anesthesia are considered if the first option is not feasible. Situation is similar in African countries and Tunisia, and activity is restricted to only urgent cases and complicated urological conditions. Diseases that are at risk of progression are performed as usual or shortly delayed, depending on the bedss/ventilators available at the time of presentation. Oncological conditions were ranked according to their importance on a surgical priority list. Each urologist is responsible for this organization according to its own perspective. There have been some discussions at national level, but for now, no proper recommendation or guidelines to be followed have been set yet.

**COVID-19 and residents learning curve:** there has been a slowdown of the residents´ learning curve in urological department since the beginning of the pandemic. Chan *et al*. [5] reported that that the pandemic caused the freezing of all interhospital staff movement, with resident shaving training in other hospitals staying there indefinitely. Puliatti *et al*. [11] stressed out on the negative impact with the delay of residency examinations, residents´ rotations and the cessation of undergraduate clinical rounds, causing high level of stress and raising concerns among residents concerning the quality of their training. In Tunisia, residents have less access to surgery room, to outpatient clinic where they learn their surgery and how to manage frequent urological conditions. Resident´s courses and congress were whether suspended or replaced by online meetings, national speciality examinations were delayed.

**Reasons behind activity drop:** reasons behind delaying urological activity are well known. In first place, there is an urgent need to provide enough material and save space for COVID units to be set up and work efficiently. Shortage of supply of personal protective equipment, intensive care units and hospital beds are a worldwide problem. In Iran, there is an excess burden of the healthcare providers due to a mismatch between COVID-19 patients requiring hospitalization and available beds in hospitals [12]. In Lombardy, Northern Italy, the surgical activity in urologic ward of Papa Giovanni hospital has completely shut down the use of available beds for patients infected with the virus [13]. In Tunisia, hospitals also suffer from a shortage of logistical resources to host infected patients. It is estimated that there are 3 intensive care beds per 100,000 inhabitants, which is far below the average number of European countries. For these reasons urological wards were emptied, decontaminated and set ready to take care of COVID patients in case of surge of hospitals beds. Medical and paramedical staff has been redeployed for the purpose of taking in charge infected patients. At each hospital, there is a parallel pathway for patients having the symptoms of the virus from the hospital entrance until the hospitalization unit. Urologists as well as other specialists contribute by seeing the patients at a separate consultation, and take care of those hospitalized, this being organized in rotation shifts. Furthermore, there is a fear of spreading the infection or being infected by the patients in case urological activity is maintained. This has been the reason why the majority of the urologists have reduced or completely stopped their work, and most of them think they are more at risk of being infected at their work rather than in their community.

## Conclusion

COVID-19 pandemic has a major impact on urological activity. Outpatient clinic and surgical activity have dramatically decreased to give space to healthcare professionals to face the pandemic. Nevertheless, we should carefully define which cases can and cannot be deferred. There is no universal definition of an ‘elective’ surgery. Recommendation for the triage of urological surgeries must be dynamic in time and re-evaluated periodically. These recommendations have to take in consideration the geographical situation and sanitary context of the pandemic. It has also to maintain a balance between the hospital burden and availability of the logistical and human resources without compromising the patient´s outcome.

### What is known about this topic

- The COVID-19 pandemic has a significant impact on the daily activity of surgical and non-surgical specialties;
- In urology, the surgical and non-surgical activity has dramatically dropped since the beginning of the pandemic;
- There is actually a need to prioritize the management of urological conditions in the context of COVID-19 pandemic.

### What this study adds

- COVID-19 had a great impact on outpatient and surgical activities in urological departments in Tunisia;
- Management of urological conditions during the pandemic is quite similar between public and private urologists except for BPH surgery;
- Most of urologists reduce their activity because of the fear of being infected by their patients/medical staff.
